# Expression profiles of circulating miRNAs in an endangered Piedmontese sheep breed during the estrus cycle

**DOI:** 10.3389/fvets.2024.1458463

**Published:** 2024-11-05

**Authors:** Isabella Manenti, Ugo Ala, Elisabetta Macchi, Irene Viola, Paola Toschi, Paolo Accornero, Mario Baratta, Silvia Miretti, Eugenio Martignani

**Affiliations:** ^1^Department of Veterinary Sciences, University of Torino, Grugliasco, Italy; ^2^Department of Chemistry, Life Sciences and Environmental Sustainability, University of Parma, Parma, Italy

**Keywords:** estrus cycle, circulating miRNA, enrichment analysis, endangered breed, sheep

## Abstract

**Introduction:**

The preservation of locally endangered breeds is essential for maintaining ecosystem services that benefit both society and the environment. Reproductive fitness becomes a crucial consideration in this context. MicroRNAs (miRNAs) are small non-coding RNA molecules that play a key role in post-transcriptional regulation. Typically, they function within the tissues where they are produced. However, when they are released into extracellular fluid, they are referred to as circulating miRNAs (c-miRNAs). C-miRNAs may serve as potential biomarkers, whose profile changes under different physiological states. The purpose of this study is to establish a connection between distinctive variations in the expression of c-miRNAs and specific estrus cycle phases in Frabosana-Roaschina sheep, an endangered Piedmontese breed.

**Methods:**

Two trials, each involving 20 ewes with different reproductive efficiencies (nulliparous in the first trial and pluriparous in the second trial), were sampled on alternate days after synchronization for blood, saliva, and feces. Ultrasound scans were performed during the induced estrus cycle. The animals’ behaviors were assessed through video recordings.

**Results:**

In the first trial, play behaviors were detected without sexual behaviors, whereas in the second trial, sexual behaviors were observed without play behaviors. Based on plasma trends of 17β-estradiol and progesterone and ultrasound images, two moments were identified for miRNAs analyses: the beginning of the follicular phase (day 2) and the beginning of the luteal phase (day 11). C-miRNAs of six representative animals from the second trial were sequenced. Analyses of the sequencing data have identified 12 c-miRNAs that were differentially expressed (DE) when comparing day 11 with day 2: five miRNAs were found to be upregulated, whereas seven miRNAs were downregulated. An enrichment analysis, based on predicted targets, using the Kyoto Encyclopedia of Genes and Genomes (KEGG) and Gene Ontology (GO) databases was performed. Many of these genes regulate reproductive pathways with the possible involvement of miRNAs. Finally, qRT-PCR was conducted to validate the DE miRNAs in all ewes. Differences in gene expression between the two sampling points and the two trials were observed, in line with existing literature.

**Discussion:**

Investigating the role of these miRNAs in regulating estrus could improve the reproductive performance and welfare of Frabosana-Roaschina ewes.

## 1 Introduction

The sheep (*Ovis aries*) is the second most bred mammal in the world, after cattle, with approximately 1.2 billion sheep (1). Due to its adaptability, gentle temperament, modest size, hardiness, and ability to use low-nutrient forage, sheep has adapted to different climatic zones and altitudes (2). Moreover, its productivity has increased in recent years (3, 4). However, both intrinsic factors, such as breed (5), and environmental and management factors, such as heat stress (6), can affect reproductive performance in sheep, highlighting the ongoing importance of reproductive efficiency and welfare issues throughout the production chain. These reproductive issues not only result in economic losses but also have a negative impact on animal welfare.

MicroRNAs (miRNAs) are small (~22 nucleotides), non-coding RNA molecules that have an important role in post-transcriptional regulation of a wide range of cellular processes, including homeostasis, immune response, and development. They are expressed at the tissue level, but when released into extracellular fluids, they are referred to as circulating miRNAs (c-miRNAs) (7). C-miRNAs may potentially serve as minimally invasive biomarkers of changes in physiological, pathological, and psychological conditions (8). To date, opportunities to comprehend the intricate physiological mechanisms driving sheep reproduction and welfare are on the rise due to recent developments in the study of circulating miRNAs. In sheep, circulating miRNAs have been shown to be associated with the reproductive sphere, such as fertility and reproductive success (9, 10). For example, Hitit et al. (11) have found variations in the expression of miRNAs in the plasma of early pregnant ewes, suggesting their potential as biomarkers for early pregnancy diagnosis.

Furthermore, miRNAs have been linked to welfare state in livestock animals, including stress and infectious diseases (12–14). The identification of miRNAs associated with stress could provide valuable insights into the physiological responses of sheep to environmental and management factors, ultimately giving strategies to improve their reproductive performance and, more generally, their welfare.

In addition to their value in livestock production systems, sheep also play a key role in preserving rural heritage and biodiversity (15). Rustic breeds, in particular, are an essential part of this heritage and are often well adapted to local conditions, requiring minimal inputs and producing high-quality meat, milk, or wool (16, 17). However, many rustic breeds are facing extinction due to the increasing popularity of high-yielding commercial breeds and the decline of traditional farming practices (18). The preservation of rustic breeds is also crucial for providing essential ecosystem services, such as conserving genetic resources, water flow regulation, pollination, climate regulation, landscape maintenance, recreation and ecotourism, and cultural heritage (19, 20). Moreover, better characterization of locally adapted breeds will be a key for adaptation breeding to address current challenges, such as climate change (21). From this perspective, the study of the reproductive lives of these breeds can be useful for their management, thus optimizing productivity and welfare.

In this study, we investigated how the signature of circulating miRNAs changed during the reproductive cycle of Frabosana-Roaschina, an Italian-endangered sheep breed of the Piedmont region. The study aimed to identify differentially expressed (DE) miRNAs that could serve as putative biomarkers to better understand the regulation of the reproductive process, thereby helping to improve the reproductive efficiency of this breed.

## 2 Materials and methods

The study approval was granted by the Italian Ministry of Health, authorization number 494/2020-PR. All methods were carried out following relevant guidelines and regulations. Informed consent is not required as no human subjects were involved in the study.

### 2.1 Ewes

The study consisted of two trials with two groups of ewes of the Frabosana-Roaschina breed. In the first trial, nulliparous ewes (10–12 months old) were recruited; in the second trial, pluriparous ewes (4–6 years old) were recruited. All ewes were moved to the sheepfold of the Department of Veterinary Sciences of Turin University. The two flocks were housed separately, away from other animals, and managed according to the breeder’s instructions. After 2 weeks of acclimatization, the sheep were synchronized with progesterone sponges for 14 days using a standard protocol (22). After the 2-week synchronization period, a ram of the same breed from the same farm was introduced into the flock. Subsequently, sample collection was carried out for 1 month (January 2020 for the first trial; February 2022 for the second trial) early in the morning and before feeding times on alternate days.

### 2.2 Ultrasound scans

On the sampling days, sheep were subjected to an ultrasound examination (Draminski Ultrasound scanner BLUE VET, linear rectal probe for sheep and goat 4,9 MHz, Poland) to assess the cyclic reproductive activity and the possible onset of pregnancy. For each ewe in the study, ultrasound images were collected every sampling day and the presence or absence of follicles and corpora lutea was evaluated.

### 2.3 Behavioral assessment

Ewes were recorded continuously using three wireless cameras (EZVIZ C3W Pro, Hangzhou Hikvision Digital Technology Co., Ltd., Hangzhou, China), located inside and outside the sheepfold, managed using a network video recorder (NVR) system (EZVIZ X5C, Hangzhou Hikvision Digital Technology Co., Ltd., Hangzhou, China). For the video analyses, a species-specific ethogram was drafted based on previous literature (Supplementary Table S1) (23). Continuous sampling of 14 days, before and during the estrous cycle of the first trial, was performed to score all behaviors expressed and identify the most commonly expressed behaviors. Subsequently, 30 min of each hour in the time slots 01–02, 10–11, and 18–19 in 3 days of three phases of the estrus cycle (first follicular phase, luteal phase, and second follicular phase) were analyzed alternately in continuous sampling. For analyses, both states and events behaviors from only “Aggressive,” “Social,” “Play” and “Sexual” categories of ethogram were considered.

### 2.4 Blood collection

Blood was collected from the jugular vein of animals using 10-mL VACUETTE® Tubes K3E K3EDTA with a VACUETTE® Multiple Use Drawing Needle 20Gx1½” (Greiner Bio-One GmbH, Kremsmünster, Austria). The plasma was separated by centrifugation at 3,500 rpm for 10 min at 5°C. Then, it was aliquoted and centrifuged again at 3,500 rpm for 10 min at 5°C to discard all cell residues. Aliquots of 1.3 mL were frozen at −80°C until processing.

### 2.5 Saliva and feces collection

Saliva samples were collected using a polyethylene pad (Salivette®, Sarstedt AG & Co., Nümbrecht, Germany). The swab was inserted into the animals’ mouths with a clamp for 3 min to allow the sheep to chew it. The saliva was collected from the swab after sampling by centrifugation at 3,500 rpm for 10 min at 5°C and stored at −20°C until processing (24–26).

Feces were collected from the rectal ampoule and stored in labeled plastic frost bags. The samples were then frozen at −20°C until processing.

### 2.6 Extraction and estimation of hormones

The quantification of plasma estradiol and progesterone concentration was performed using commercial ELISA kits (DetectX® 17β-Estradiol ELISA Kit and DetectX® Progesterone ELISA Kit; Arbor Assays®, USA) to detect ewes’ estrus cycle. Hormones were extracted from the plasma using diethyl ether, following the Steroid Liquid Sample Extraction Protocol for DetectX® Immunoassay Kits (Arbor Assays®, USA).

Cortisol concentration was determined from both saliva and fecal samples of all ewes.

To extract cortisol from the feces, these were left to dry on a stove at 65°C for 2 days. Then, dried feces were powdered and an aliquot of 250 mg was weighed. For extraction, 3 mL of diethyl ether was added to each aliquot and vortexed for 3 min. After 1 h at −20°C to freeze the solid component, the ether was collected in glass extraction tubes. The tubes were left overnight in a fume hood until complete evaporation of diethyl ether. When estimating cortisol levels, the samples were resuspended in 1 mL of ethanol, and subsequently, 1 mL of PBS was added.

For both salivary and fecal cortisol quantification, an ELISA sandwich immunoassay has been performed using a commercial ELISA cortisol kit (DetectX® Cortisol ELISA Kit; Arbor Assays®, USA) according to the manufacturer’s protocol.

### 2.7 Extraction and quantification of plasma microRNAs

The extraction process was performed starting from 500 μL of centrifuged plasma using the Maxwell® RSC miRNA Plasma or Serum kit (Promega, Madison, Wisconsin, USA) according to the manufacturer’s protocol. During the extraction process, 1 μL of UniSp2,4,5 spike-in (Qiagen, Hilden, Germany) was added to each sample as a quality control measure.

miRNA quantification was performed using a Quantus™ Fluorometer (Invitrogen, Thermo Fisher Scientific Inc., Waltham, Massachusetts, USA) and a Qubit™ microRNA Assay Kit, following the manufacturer’s guidelines. The miRNA samples were then stored at −80°C.

### 2.8 Sequencing

Six ewes were randomly chosen, and for each animal, two time points were selected (day 2 and day 11 after the end of synchronization). Therefore, a total of 12 RNA samples were selected and quality tested using the Agilent 2100 Bioanalyzer RNA assay (Agilent Technologies, Santa Clara, California, USA). Then, they were sent for sequencing using an external service (IGA Technology Services, Udine, Italy).

For the preparation of libraries, the QIAseq miRNA Library Kit (Qiagen, Hilden, Germany) was used following the manufacturer’s instructions. The final libraries were checked using both Qubit 2.0 Fluorometer (Invitrogen, Carlsbad, California, USA) and Agilent Bioanalyzer DNA assay or using the Caliper LabChip GX (PerkinElmer, Waltham, Massachusetts, USA). The libraries were then prepared for sequencing and sequenced on single-end 150-bp mode on NextSeq 500/NovaSeq 6000 (Illumina, San Diego, California, USA).

Both raw read counts and normalized reads (per million of the total number of reads in the analysis) were provided.

### 2.9 cDNA synthesis and quantitative real-time qPCR

Immediately after extraction and quantification, 0.8 μL of miRNA samples were reverse transcribed in cDNA using a miRCURY LNA™ RT Kit (Qiagen, Hilden, Germany) in a final volume of 10 μL according to the manufacturer’s instructions. At this stage, 0.5μL of UniSp6 spike-in (Qiagen, Hilden, Germany) was added to each sample as quality control for reverse transcription.

The cDNA samples were stored at −20°C until analysis.

Before RT-qPCR, the cDNA was diluted 30-fold before use, and amplification was carried out using a miRCURY LNA™ SYBR Green PCR Kit according to the manufacturer’s protocol (Qiagen, Hilden, Germany). The PCR cycling conditions consisted of an initial heat activation at 95°C for 2 min, followed by a two-step cycling for 40 times: denaturation at 95°C for 10 s and combined annealing and extension at 56°C for 60 s. Melting curve analyses were performed between 60 and 95°C.

All primers of miRNAs used in RT-qPCR are miRCURY LNA miRNA PCR Assay (Qiagen, Hilden, Germany). The references of the analyzed miRNAs are given in Table 1.

**Table 1:** Designation of microRNAs.

miRNA name	Primer ID	Qiagen GeneGlobe ID
miR-221	cfa-miR-221	YP02104713
miR-26b	hsa-miR-26b-5p	YP00204172
miR-103	oar-miR-103	YP02110471
miR-432	hsa-miR-432-5p	YP00204776
let-7c	hsa-let-7c-5p	YP00204767
miR-493-3p	hsa-miR-493-3p	YP00204557
miR-376b-3p	oar-miR-376b-3p	YP02100887
miR-143	hsa-miR-143-3p	YP00205992
miR-22-3p	oar-miR-22-3p	YP02114472
miR-10b	oar-miR-10b	YP02102190
miR-23a	oar-miR-23a	YP02103289
miR-150	hsa-miR-150-5p	YP00204660
miR-133	dme-miR-133-3p	YP00205954
miR-16b	oar-miR-16b	YP02115941
miR-665-3p	oar-miR-665-3p	YP02114331
miR-369-3p	hsa-miR-369-3p	YP00206028
miR-30a-5p	oar-miR-30a-5p	YP02106730
miR-27a	hsa-miR-27a-3p	YP00206038
miR-494-3p	rno-miR-494-3p	YP02112391
miR-1185-3p	oar-miR-1185-3p	YP02116317

### 2.10 Statistical analyses

Differentially expressed (DE) miRNAs were identified from next-generation sequencing data using edgeR (version 4.2.0). Subsequent functional analysis was conducted with clusterProfiler (version 4.8.3), DOSE (version 3.26.2), org.Bt.eg.db (version 3.17.0), and org.Hs.eg.db (version 3.17.0) packages in R (version 4.3.3) within the RStudio (release 2023.06.1) environment.

Since there is no archive for *Ovis aries*, orthologous miRNAs were identified using miRBase^1^ for *Homo sapiens* and *Bos Taurus.* Genes and transcripts putatively regulated by DE miRNAs were selected using the TargetScan Release 8.0 databases. Only those with at least one conserved 8mer site were included.

An enrichment analysis was then carried out on these genes using databases such as Gene Ontology (GO) and Kyoto Encyclopedia of Genes and Genomes (KEGG).

From the RT-qPCR results, stable miRNA normalizers were identified using qbase+ software (Biogazelle, Zwijnaarde, Belgium^2^) (27), and their geometric mean was calculated. For statistical analyses of the RT-qPCR data, ∆Cq was used, defined as the difference between the Cq of the miRNA target and the geometric mean of normalizers’ Cq.

To validate the differentially expressed miRNAs across all sampled animals, we performed Wilcoxon and Mann–Whitney tests, as well as paired-and independent-samples Student’s *t*-tests. Normality assumptions were verified using the Shapiro–Wilk and Kolmogorov–Smirnov normality tests for each sampled time point, in both trials, using GraphPad Prism version 9.0.0 for Windows (GraphPad Software, Boston, Massachusetts, USA^3^).

## 3 Results

### 3.1 Reproductive status

**Figure 1:**
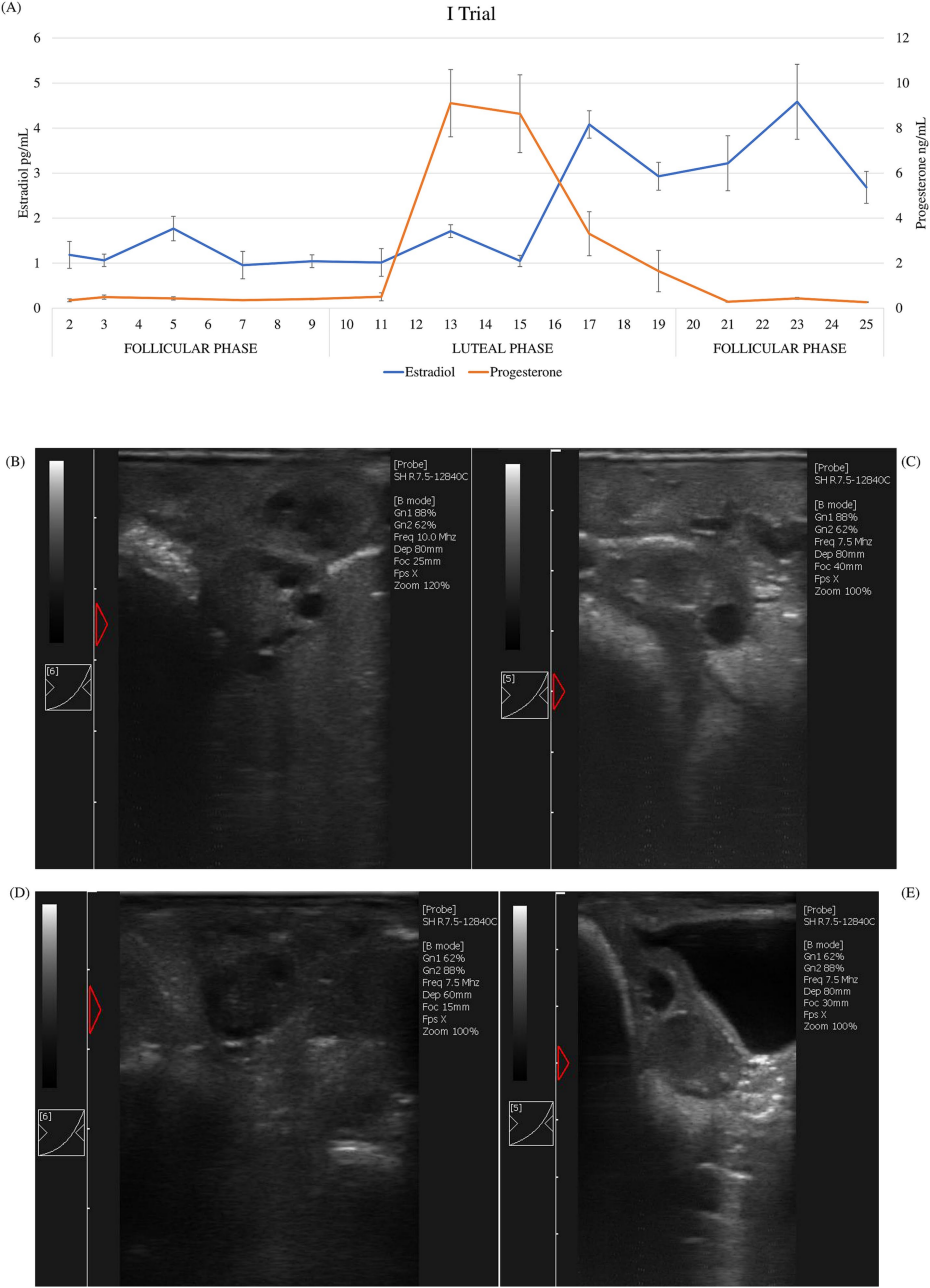
First trial: **(A)** average of estradiol and progesterone concentrations (±SD) of ewes (*n* = 20) and ultrasound scan images of a representative ewe: **(B)** growing follicles at day 2; **(C)** dominant follicle at day 9; **(D)** early corpus luteum at day 11; **(E)** corpus luteum at day 15.

**Figure 2:**
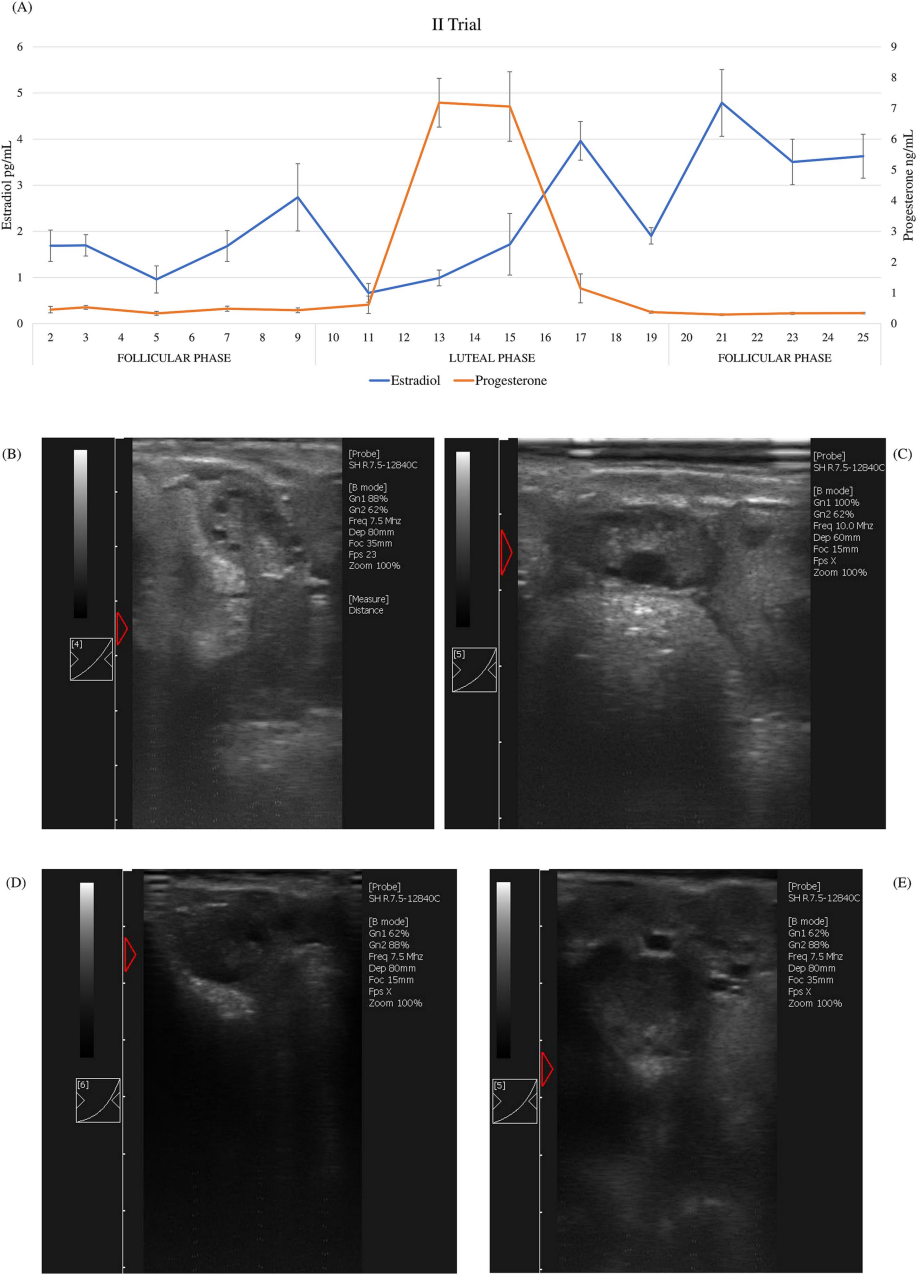
Second trial: **(A)** average of estradiol and progesterone concentrations (±SD) of ewes (*n* = 20) and ultrasound scan images of a representative ewe: **(B)** growing follicles at day 2; **(C)** dominant follicle at day 9; **(D)** early corpus luteum at day 11; **(E)** corpus luteum at day 15.

According to hormonal patterns (estradiol and progesterone) during the sampling period, the expected cyclicity was displayed. During the estrus cycle in both trials, average estradiol value concentrations ranged between 0.96 and 4.79 pg/mL, whereas progesterone ranged between 0.26 and 9.11 ng/mL. Different phases of the estrus cycle were identified (Figure 1A and Figure 2A). In both trials, the luteal phase was clearly evident: progesterone peaked on the 12th day of the cycle, maintained a plateau until the 15th day, and then dropped back to the baseline levels on the 21st day. Peaks of estradiol, associated with follicular waves, appeared after the progesterone peak: on the 17th and 23rd day in the first trial and on the 17th and the 21st day in the second trial between the two cycles. Additionally, a significant increase in estradiol (*p* < 0.01) was observed on day 9, prior to the luteal phase, in the second trial. These data show that the estrus cycle of Frabosana-Roaschina ewes lasted approximately 18 days.

Additionally, the animals’ ovarian cyclicity was validated by ultrasound scan images. Follicular growth was detected during the follicular phase, which was identified based on hormone plasma concentration. Corpora lutea were detected after the peak of progesterone plasma concentration (Figures 1B–E, 2B–E).

When comparing behaviors, differences were evident between the two trials. Ewes in the first trial showed only several play behaviors but no sexual behaviors, such as clear manifestations of estrus (Table 2). During the second trial, however, sexual behaviors were displayed by ewes and no play behaviors were registered (Table 3).

**Table 2:** List of behaviors recorded during first trial. Several play behaviors, in addition to aggressive and affiliative behaviors, but no sexual behaviors were registered.

**Aggressive and threatening behaviors**	**Social and affiliative behaviors**	**Play behaviors**
Butt	Pushing	Gambole
Head clash	Displacing from resources	Chasing
Blocking	Nudging	Reciprocal butt
Threaten	Sniffing	Jumping
Horn threat (jerk)	Nose	Frontal butt (one-way butt)
Threat kick	Grooming	Side/rear butt (one-way butt)
Horn and shoulder pushing	Licking	Racing
	Mount	
	Kick	

**Table 3:** List of behaviors recorded during the second trial. In addition to aggressive and social or affiliate behaviors, sexual behaviors were recorded.

**Aggressive and threatening behaviors**	**Social and affiliative behaviors**	**Sexual female behaviors**
Butt	Pushing	Squat/crouch
Blocking	Displacing from resources	Head turning
Threaten	Nudging	Standing mount
Horn threat (jerk)	Sniffing	Following/migration
Threat kick	Nose	
	Grooming	
	Licking	
	Mount	
	Kick	

Based on hormonal trends, ultrasound images, and behavioral assessments, it can be assumed that the ewes in the first trial exhibited silent ovulation.

Finally, during the same experimental period, salivary cortisol levels ranged from 0.64 to 2.01 ng/mL, whereas fecal cortisol ranged from 8.64 to 19.66 ng/g. No statistical significance was highlighted in the cortisol values between the follicular phase and the luteal phase. The statistical analyses did not reveal any significant differences in the cortisol trends during the sampling period in either matrix.

### 3.2 Sequencing data show DE miRNA and related downstream pathways

Based on the hormonal patterns of estradiol and progesterone and on ultrasound scan images, two sampling points were chosen: day 2 after the end of synchronization, and day 11, immediately before the progesterone rise. The two sampling points therefore correspond to the beginning of the follicular phase and the beginning of the luteal phase respectively, coherently with the ovaries’ ultrasound scans. Plasma miRNAs were extracted from six selected animals from the second trial at both time points and sequenced. For sequencing, animals from the second trial were chosen for their reproductive competence compared to animals in the first trial, in order to investigate any possible changes linked to ovarian cyclicity in the expression patterns of the circulating microRNAs.

#### 3.2.1 Most expressed microRNAs

Once data were normalized (reads per million, RPM) for each sequenced sample, we chose the 10 miRNAs with the highest read count. In all samples, a subset of seven miRNAs (oar-miR-16b, −191, oar-let-7a, −7b, −7f, −7g, and –7i) was shared as highly expressed, at all sampling points. Seven out of the 12 samples also shared a subset of three miRNAs (oar-miR-26b, −29a, and −103) among the most expressed.

#### 3.2.2 Differentially expressed (DE) microRNAs during estrus

Data analysis revealed that 12 miRNAs were differentially expressed (nominal *p*-value <0.01) between the two sampling points. Only miRNAs expressed in at least two-thirds of the sequenced samples, with average and maximum read values exceeding 10, were considered. Comparing day 11 with day 2, five miRNAs were upregulated (oar-miR-103, −376b-3p, −432, −493-3p, and oar-let-7c), and seven were downregulated (oar-miR-10b, −22-3p, −23a, −27a, −133, −143, and −150). Then, a list of highly stable miRNAs in all samples was compiled, and the two least variables (mir-26b and −221) were selected as housekeeping miRNAs for subsequent gene expression analysis through qPCR.

#### 3.2.3 Enrichment analysis

Putative targets of these DE miRNAs identified using TargetScan were subjected to an enrichment analysis. The functional analysis showed a wide variety of pathways (Supplementary Figures S1A,B, S2A–C). Among these pathways, we have identified several that were related to reproductive activity and stress response (with the number of genes involved in each pathway indicated in parentheses). When analyzing *Bos taurus* putative targets after querying with downregulated miRNA orthologs, we found differences in the following biological processes (BP) from the GO database: rhythmic process (12), response to hormone (20), response to endogenous stimulus (35), and insulin receptor signaling pathway (6). For the KEGG database, the affected pathways included insulin signaling pathway (24), insulin resistance (20), prolactin signaling pathway (15), estrogen signaling pathway (23), circadian rhythm (9), endocrine resistance (17), oxytocin signaling pathway (24), and MAPK signaling pathway (49). In the *Homo sapiens* database, we isolated cellular response to endogenous stimulus (178), behavior (87), regulation of circadian rhythm (60), rhythmic process (47), mammary gland development and morphogenesis (40), regulation of MAPK cascade (92), cellular response to hormone stimulus (176), miRNA metabolic process (18), miRNA transcription (15), positive regulation of miRNA metabolic process (12), cellular response to stress (189), positive regulation of miRNA transcription (11), activin receptor signaling pathway (10), hormone-mediated signaling pathway (27), cellular response to steroid hormone stimulus (29), and behavioral fear response (9) as pathways emerged when querying with downregulated miRNAs human orthologs. Cellular response to stress (138), regulation of cellular response to stress, regulation of translational initiation in response to stress, and integrated stress response signaling (62) emerged when querying with upregulated miRNAs human orthologs in biological processes (BP) in the GO database. Interesting downregulated human miRNA orthologs resulted in putatively affecting molecular functions (MF) in the GO database, such as nuclear steroid receptor activity (9), nuclear glucocorticoid receptor binding (11), activin binding (4), and nuclear estrogen receptor binding (9). Circadian rhythm (9), insulin resistance (30), GnRH signaling pathway (10), and MAPK signaling pathway (40) were found with the same query in the KEGG database.

### 3.3 Gene expression analysis of circulating miRNAs

To expand on the DE miRNA analysis, we performed a gene expression analysis through qPCR on all 40 animals (20 of the first trial and 20 of the second trial), statistically comparing the expression profiles of miRNAs between the two selected sampling points (day 2 and day 11). In addition to the differentially expressed miRNAs that emerged from sequencing, oar-miR-16b, the most expressed, was also analyzed. Moreover, 3 miRNAs (oar-miR-30a-5p, −369-3p, and −665-3p) from the literature were included in the analysis for their possible involvement in reproduction and stress pathways.

**Figure 3:**
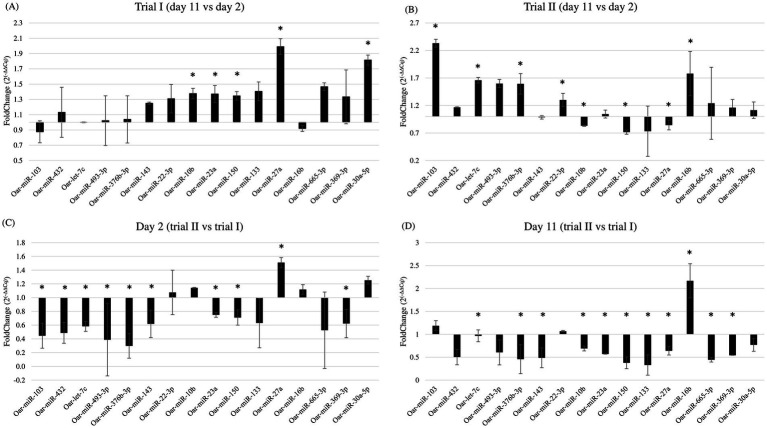
Fold change of miRNAs expression comparing **(A)** day 11 with day 2 in the first trial; **(B)** day 11 with day 2 in the second trial; **(C)** day 2 of the second trial with the day 2 of the first trial; **(D)** day 11 of the second trial with day 11 of the first trial. * Indicates statistically significant differences in miRNA expression between trials or days. Individual *p*-values are reported in the main text.

For every miRNA considered, amplification data from RT-qPCR were normalized using the geometric mean of oar-miR-26b, −221, the two miRNAs that, from sequencing data analysis, showed the least variation in their expression across samples, and UniSp6, a control spike-in (in parentheses, *p*-value, and fold change of every miRNA). In the first trial, oar-miR-10b (*p* < 0.05; 1.38), −23a (*p* < 0.01; 1.37), −27a (*p* < 0.01; 1.99), −30a-5p (*p* < 0.01; 1.82), and −150 (*p* < 0.01; 1.35) resulted significantly upregulated at day 11 (Figure 3A). In the second trial, we found that at day 11 there was a significant upregulation of oar-let-7c (*p* < 0.01; 1.66), oar-miR-16b (*p* < 0.01; 1.78), −22-3p (*p* < 0.01; 1.30), −103 (*p* < 0.01; 2.33), and -376b-3p (*p* = 0.017; 1.59), whereas oar-miR-10b (*p* = 0.037; 0.83), −27a (*p* = 0.013; 0.84), and − 150 (*p* < 0.01; 0.72) were downregulated (Figure 3B). When comparing the same sampling point between the two trials, a statistically significant difference for several miRNAs was found. When comparing the expression of miRNAs between the two trials on day 2 (second vs. first), we found that oar-let-7c (*p* < 0.01; 0.59), oar-miR-23a (*p* < 0.01; 0.75), −103 (*p* < 0.01; 0.45), −143 (*p* < 0.01; 0.62), −150 (*p* = 0.018; 0.71), −369-3p (*p* < 0.01; 0.62), −376b-3p (*p* < 0.01; 0.3), −432 (*p* < 0.01; 0.49), and −493-3p (*p* < 0.01; 0.39) were downregulated, whereas oar-miR-27a (*p* < 0.01; 1.51) was upregulated (Figure 3C). When comparing the second time point, day 11, we found the following miRNAs were downregulated in the second trial: oar-miR-10b (*p* = 0.013; 0.69), −23a (*p* < 0.01; 0.57), −27a (*p* < 0.01; 0.64), −133 (*p* < 0.01; 0.33), −143 (*p* < 0.01; 0.49), −150 (*p* < 0.01; 0.38), −369-3p (*p* < 0.01; 0.54), −376b-3p (*p* < 0.01; 0.46), −432 (*p* = 0.015; 0.5), and 665-3p (*p* < 0.01; 0.44). In contrast, oar-miR-16b (*p* < 0.01; 2.17) was the only miRNA that was resulted upregulated (Figure 3D).

Hence, when confronting the two trials, the only two miRNAs upregulated on day 2 and day 11 were, respectively, oar-miR-27a and oar-miR-16b, whereas oar-miR-23a, −143, −150, −369-3p, −376b-3p, and −432 were downregulated for both days in the second trial.

When considering the second trial, which provided the samples that underwent sequencing, six miRNAs (oar-let-7c, oar-miR-10b, −27a, −103, −150, and -376b-3p) showed variations in expression that were concordant with the fold changes reported in the analysis of the sequencing data, one miRNA (oar-miR-22-3p) showed an opposite trend compared to the sequencing data analysis, whereas five miRNAs (oar-miR-23a, −133, −143, −432, and − 493-3p) were found to be not differentially expressed.

## 4 Discussion

### 4.1 Nulliparous 10–12-month ewes of the Frabosana-Roaschina breed showing reproductive immaturity

Frabosana-Roaschina is an endangered sheep breed of the North Western Alps, specifically the Piedmont region. It is an autochthonous breed of medium size, rustic and frugal, with a typically snub profile and flattened horns, spiraled in the males and facing backward in the females, which has a marked aptitude for milk production. The breeding system is mostly transhumant, with summer alpine pasture and winter housing (28). We synchronized them to ensure that every ewe was in the same phase at the same time, and based on hormone patterns and ultrasound images, we considered that the ewes had an estrus cycle of approximately 18 days. The length of the cycle is similar to that of the other sheep breeds and coherent with what has been reported in the literature (29). According to the protocols of synchronization with progesterone sponges, in sheep, the estrus should begin approximately 48 h after device removal (30). In our study, through hormonal trends and ultrasound images, we found that ovulation occurred later than expected. From the data obtained, we can identify a follicular phase up to day 9, and a luteal phase from day 10 to day 19. Wildeus (22) observed that estrus response and fertility vary greatly when intravaginal sponges are applied, dependent on species, breed, co-treatment, management, and mating system. This may be due to gonadotropic insufficiency and low ovarian response of the rustic breed (31). In the second trial, prior to the increase and plateau of progesterone, we observed a significant peak of estradiol that was not present in the first trial. This might be because the nulliparous ewes that were considered in the first trial were not reproductively competent yet, unlike the ewes recruited in the second trial. We have selected the sampling points (day 2 and day 11) based on hormonal data and ultrasound images, that confirmed ovarian status. We selected day 2, when we observed multiple smaller follicles in the ovary, as the beginning of the follicular phase. Considering that we detected the peak of progesterone on day 13 following sponge removal, and observed dominant follicles on ultrasound images at day 9 and a corpus luteum at day 15, we chose day 11 as the sampling point to mark the beginning of the luteal phase.

Moreover, it was observed that the ewes in the first trial ovulated without exhibiting estrus (“silent ovulations”), despite the treatment with progesterone sponges for 14 days, which is adequate to trigger the estrus (22). The fact that no reproductive behavior was seen in the first trial provides evidence of this. Quirke (32) revealed that during the typical onset of the breeding season, the incidence of silent ovulations is higher in lambs than in adult ewes. It is also possible that this feature was impacted by the fact that nulliparous ewes were housed separately, in a group, and distant from more mature sheep, which may have limited their experience and prevented them from learning from adult sheep (33). In addition, Santos-Jimenez et al. (34) and Ungerfeld et al. (35) reported that nulliparous sheep failed to develop estrous behavior after progesterone removal. Therefore, our data confirm that the reproductive response of yearling lambs is not fully developed compared to that of mature ewes.

Regarding the cortisol patterns in saliva and feces, no statistically significant variations were seen in either of the two trials, even though in literature, several studies report changes in cortisol levels in saliva and feces following acute and chronic stressors (36–39).

### 4.2 Enrichment analysis shows pathways generally related to the reproductive sphere

When enrichment analyses were performed, putative targets of downregulated miRNAs were involved in several pathways shared between *Bos taurus* and *Homo sapiens*. Some of these were more general, but fit with the scope of our hypothesis: response to hormones, response to endogenous stimulus, rhythmic process, and circadian rhythm. Other shared pathways were more closely related to hormonal processes that we considered, such as estrogen and steroid hormones signaling and response, insulin signaling pathway and resistance, and MAPK (mitogen-activated protein kinase) regulation and signaling pathway. Jansen et al. (40) found that the expression of ERβ (estrogen receptor beta) mRNA over granulosa cells declined with increasing follicle size in sheep. From ultrasound images, we detected growing follicles between day 2 and day 11. In light of this, the changes in estrogen-related pathways that we highlighted are in accordance with evidence from the literature. Insulin is a peptide hormone whose antagonist is cortisol, among others, and is also involved in stress response. Hormones that, to a lesser extent, stimulate insulin secretion are estrogens, which, in turn, stimulate the synthesis of transcortin, a protein that binds and inhibits cortisol (41, 42). Freitas-de-Melo et al. (43) observed that, in sheep, the physiological and behavioral responses changed according to the phase of the estrous cycle. They report that serum total protein, globulin, and plasma glucose concentrations were greater during the follicular phase when compared to the luteal one. Insulin has regulatory effects on glucose and globulins, such as sex hormone-binding globulin (SHBG) (44). The results of these studies are coherent with the putative-affected pathways that emerged from our enrichment analyses.

The pathways that emerged from the putative targets of miRNAs that were downregulated only in *Bos taurus* include prolactin and oxytocin signaling pathways. The fact that their signaling pathways were significantly affected by downregulated miRNAs between the two phases of the ovarian cycle is consistent with their physiological function.

In the *Homo sapiens* database, several pathways related to the regulation and transcription of miRNAs are of interest. Other pathways relate to hormones, such as activin and GnRH, which are involved in ovulation. A downregulation of activin could lead to a decrease in SMAD2 activity. This agrees with the upregulation that resulted between day 2 and day 11 of miR-16b, which reduces SMAD protein levels in sheep (45).

Behavior and behavioral fear response are two pathways that emerged from the enrichment analysis when we examined the *Homo sapiens* orthologs. Such evidence might be important in trying to understand how c-miRNAs might be involved in changes in behaviors like the ones we observed between the two trials, specifically in play and sexual behaviors.

Finally, there are two pathways for *Homo sapiens* orthologs that are associated with significantly upregulated miRNAs: cellular stress response and regulation of cellular response to stress, regulation of translational initiation in response to stress, and integrated stress response signaling. It is fascinating that cellular stress response has also emerged among pathways linked to downregulated miRNAs. These data highlight the multiplicity of genes involved and the complexity in the miRNAs regulation of pathways related to stress, even though we could not detect significant differences in cortisol concentrations in both analyzed matrices.

As we find consistency between the differences detected in our research and the pathways that emerged from the expression analysis of miRNAs, we can speculate that differentially expressed miRNAs can directly regulate these pathways.

### 4.3 Gene expression analysis of circulating miRNAs

The validation of DE miRNAs using RT-qPCR confirmed the trends of sequencing analysis for most miRNAs.

Oar-let-7c belongs to a family of highly conserved miRNAs across species (46, 47). Let-7 is the miRNA cluster that is most expressed in ovary (48). De Los Reyes et al. (49) found miR-let-7c is downregulated during the estrus phase in dogs, when an increased expression of ESR2, the gene coding estrogen receptor beta (ERβ), which is a putative target of miR-let-7c, is evident. These data agree with the presence of miR-let-7c in the ovine (50) and caprine (51) granulosa cells (GCs). In our study, we detected a significant upregulation at day 11 of oar-let-7c in the second trial. Considering hormonal trends, this increase of miRNA is consistent with literature data.

The effects of the miR-10 family, which includes miR-10a and miR-10b, on the proliferation and apoptosis of granulosa cells in humans, rats, and mice are highly conserved (52, 53). Here, we found that oar-miR-10b is significantly lower on day 2 in the first trial. This agrees with the results of Peng et al. (54), who found that, in goat, miR-10b inhibits granulosa cell proliferation by targeting BDNF (brain-derived neurotrophic factor). Similar results were obtained by Li et al. (55) in porcine species, where miR-10b inhibits the CYP19A1 gene, required for estradiol synthesis and prevention of cell apoptosis in porcine GCs. In the second trial, we observed a trend opposite to that of the first trial, with a downregulation between the two selected points. Further studies will be needed to clarify the role of this miRNA, probably associated with the estrus cycle and, more broadly, reproductive efficacy.

Oar-miR-16b is the most expressed miRNA in all our 12 samples, and in the literature, it is linked to granulosa cell proliferation in polycystic ovarian syndrome in humans (56) and related to stress response in sheep (12). The interesting evidence is that, in porcine species, Gad et al. (57) found miR-16b among the top 10 highly abundant miRNAs in porcine oocytes.

Another miRNA upregulated at day 11, in the second trial, is oar-miR-22-3p. In sheep, this miRNA is found more expressed in the oviduct and ovaries during the luteal phase when compared to the follicular phase (58, 59). Moreover, its overexpression leads to a reduction in the ER*α* levels (60). This is in line with our qPCR results, in which we observed an increase in the expression of miR-22 at the approach of the luteal phase.

We also identified oar-miR-23a upregulated at day 11 of the first trial and downregulated at both time points when comparing the second trial to the first. In the literature, miR-23a is associated with apoptosis of granulosa cells in humans (61–63) and in yaks (64), it is also heavily involved in the hormonal reproductive axis by modulating ER-α and ER-*β* expression, estradiol and progesterone concentrations. Other studies performed on dogs (65) and horses (66) link this miRNA to age reproductive competence.

miR-27a belongs to the same cluster of miR-23a. In our study, we found two opposite trends between the first and the second trial: upregulated in the first one and downregulated in the second at day 11. In the literature, several mechanisms of action are reported for this miRNA. In humans, it promotes apoptosis in granulosa cells (62), and it is upregulated in the luteal phase in sheep (58). These data appear to agree better with the trend we found in the first trial. In other studies, it is found downregulated in the luteal compared to follicular ovaries of sheep (59) and downregulated in large follicles compared to small follicles in sheep (67). These data, on the contrary, match our trend in the second trial. Such difference might be due to the specific breeds analyzed.

Oar-miR-103 is one of the miRNAs upregulated at day 11 in the second trial. It has been studied for its involvement in buffalo progesterone-mediated oocyte maturation and pregnancy (68). Moreover, miR-103 has been shown by Sirotkin et al. (53) to influence estradiol and progesterone secretion in human ovarian cells.

Oar-miR-143 was downregulated in the second trial vs. the first one at both time points. In the literature, miR-143 has been reported as a negative regulator of the FSH signaling pathway and to reduce estradiol secretion in mice (69). It is upregulated in monotocous sheep during the luteal phase affecting GnRH release (70), it inhibits steroidogenesis and induces apoptosis in porcine granulosa cells (71) and it affects both the synthesis of progesterone and the secretion of estradiol, reducing it in the bovine species (72). The variations we observed in this c-miRNA concentration, noted only when comparing the two trials, may suggest a role in the synthesis and regulation of progesterone and estradiol associated with the development of reproductive competence, rather than modulating the estrus cycle.

Oar-miR-150 is significantly downregulated on day 11 of the second trial and at both sampling points when comparing the second trial with the first one. Zhang et al. (73) described miR-150 as a possible regulator of synaptic plasticity of the hippocampus that plays a significant role in stress-induced anxiety-like behavior in adult mice. It is interesting its increase in sheep ovaries during the follicular phase compared to luteal, targeting the STAR gene and, subsequently, acting on steroidogenesis (74). This is consistent with the trends we observed in the second trial. Moreover, Geng et al. (75) found that miR-150 is a negative control of the production of sex-steroid precursor, regulating negatively the expression of STAR protein and steroidogenesis. We can hypothesize that the downregulation of this miRNA, which acts by reducing the precursors of sex steroids, removes a repressive stimulus on the synthesis of sexual hormones in pluriparous adult animals.

In our study, oar-miR-376b-3p was found upregulated at day 11 in the second trial and downregulated on both day 2 and day 11 when comparing the second trial with the first one. Data about miR-376b linked to stress and reproduction are scarce, but it is specifically expressed in adrenal tissue in sheep under different photoperiod treatments, consequently influencing the regulation of estrus (76). Song et al. (59) found miR-376c (a member of the same family) upregulated in the luteal phase compared to follicular one. This agrees with the trend we detected in the second trial.

Finally, we noticed a downregulation for both sampling points in the second trial compared to the first one for oar-miR-432. This miRNA is expressed in the ovaries of Hanper sheep (77) and may be related to the release of GnRH hormone, thereby affecting the reproductive performance of sheep (78). Additionally, it downregulates RPS6KA1 expression, a gene that regulates ovarian development in the small-tail Han sheep (79).

After analyzing the DE miRNAs based on sequencing data, we included three more miRNAs from the literature. Oar-miR-30a-5p was significantly upregulated on day 11 in the first trial. It appears to be related to inhibition of granulosa cell death in chickens (80), and is also involved in heat stress response (81, 82) and depression vulnerability (83). This miRNA may be more involved in regulating stress response, rather than directly affecting reproduction-related pathways. Oar-miR-369-3p is downregulated for both day 2 and day 11 in the second trial compared to the first. Oar-miR-665-3p is downregulated only for day 11 in the second trial. According to recent studies, miR-369 is linked to progesterone levels (84) and inhibits progesterone production in the bovine species (85). miR-665 is highly expressed in the luteal tissues of buffalo (86), regulates luteal function, and is a positive regulator of the lifespan of the corpus luteum in small ruminants (87). The fact that we find it lower in the second trial may be the effect of animal reproductive efficiency.

In conclusion, we wanted to describe and study the changes in c-miRNAs associated with specific phases of the reproductive cycle of a rustic endangered breed of sheep through the use of different resources. Several studies, in addition to those mentioned in this article, have investigated the identification and expression of circulating and tissue miRNAs related to reproductive performance in sheep (88–90). By comparing the two trials, during which ewes of the same breed were at different stages of their reproductive lives, we aimed to provide insights that could be useful in the management of the breed, thus optimizing its productivity and welfare.

Among miRNAs that emerged from this study, oar-miR-432 was also found in sheep ovaries related to reproductive traits, such as GnRH release (70), fertility (88), prolificity (90), and estrus during the non-breeding season (91). For this reason, it could be a promising biomarker of reproductive competence and health in sheep. Other interesting miRNAs involved in sheep estrus are oar-miR-143 (10, 70, 92), −103 (88, 91), −376b-3p (89, 91), and -493-3p (89, 91). Finally, oar-miR-10b has been reported as the most expressed by some authors (90, 91), whereas others reported it as differentially expressed (10, 88, 92). It is clear that these miRNAs still need further study to better define their role in the reproduction of sheep and, more specifically, in the Frabosana-Roaschina breed. When considering the significant differences in the expression of selected circulating miRNAs and the putative pathways that they might regulate, the overall picture shows interesting ties to the hormonal reproductive axis. In the literature, there are numerous reports that support the idea that the miRNAs that we identified might affect the functions of different endocrine glands of the HPO axis. A specific plasma c-miRNA signature might be associated with a complex regulatory network and might contribute to coordinating the sequence of the phases of the reproductive cycle.

Finally, we believe that this study may be important in a changing livestock breeding landscape, where the improvement of local breeds and the enhancement of local products for the better welfare of the entire production chain is increasingly gaining the spotlight.

## Data Availability

The data presented in the study are deposited in the Gene Expression Omnibus (GEO-NCBI, https://www.ncbi.nlm.nih.gov/geo/) repository, accession number GSE271102.

## Footnotes

^1^ https://www.mirbase.org/

^2^ www.qbaseplus.com

^3^ www.graphpad.com

## References

[1] Food and Agriculture Organization, FAO statistical yearbook 2021. FAOSTAT, stat. Database (2021) Available at: https://www.fao.org/faostat/en/#data/QCL (Accessed June 06, 2024)

[2] Simões J Abecia JA Cannas A Delgadillo JA Lacasta D Voigt K. Review: managing sheep and goats for sustainable high yield production. Animal. (2021) 15:100293. doi: 10.1016/j.animal.2021.100293, PMID: 34294548

[3] Robinson TP Wint GRW Conchedda G Van Boeckel TP Ercoli V Palamara E. Mapping the global distribution of livestock. PLoS One. (2014) 9:e96084. doi: 10.1371/journal.pone.0096084, PMID: 24875496 PMC4038494

[4] Gilbert M Nicolas G Cinardi G Van Boeckel TP Vanwambeke SO Wint GRW. Global distribution data for cattle, buffaloes, horses, sheep, goats, pigs, chickens and ducks in 2010. Sci Data. (2018) 5:1–11. doi: 10.1038/sdata.2018.227 30375994 PMC6207061

[5] Heinzen BC Weber SH Milczewski V Maia D Kozicki LE Sotomaior CS. Reproductive performance of European-breed ewes in different seasons of the year under mid-latitude. Reprod Domest Anim. (2023) 58:740–5. doi: 10.1111/rda.14344, PMID: 36920259

[6] van Wettere WHEJ Kind KL Gatford KL Swinbourne AM Leu ST Hayman PT. Review of the impact of heat stress on reproductive performance of sheep. J. Anim. Sci. Biotechnol. (2021) 12:26. doi: 10.1186/s40104-020-00537-z, PMID: 33583422 PMC7883430

[7] O’Brien J Hayder H Zayed Y Peng C. Overview of Micro RNA biogenesis, mechanisms of actions, and circulation. Front Endocrinol. (2018) 9:402. doi: 10.3389/fendo.2018.00402, PMID: 30123182 PMC6085463

[8] Do DN Dudemaine PL Mathur M Suravajhala P Zhao X Ibeagha-Awemu EM. miRNA regulatory functions in farm animal diseases, and biomarker potentials for effective therapies. Int J Mol Sci. (2021) 22:3080. doi: 10.3390/ijms22063080, PMID: 33802936 PMC8002598

[9] Miao X Luo Q Zhao H Qin X. Ovarian transcriptomic study reveals the differential regulation of miRNAs and lncRNAs related to fecundity in different sheep. Sci Rep. (2016) 6:35299. doi: 10.1038/srep35299, PMID: 27731399 PMC5059661

[10] Yang J Li X Cao YH Pokharel K Hu X Chen ZH. Comparative mRNA and miRNA expression in European mouflon (*Ovis musimon*) and sheep (*Ovis aries*) provides novel insights into the genetic mechanisms for female reproductive success. Heredity. (2018) 122:172–86. doi: 10.1038/s41437-018-0090-1, PMID: 29784930 PMC6327046

[11] Hitit M Kose M Kaya MS Kırbas M Dursun S Alak I. Circulating miRNAs in maternal plasma as potential biomarkers of early pregnancy in sheep. Front Genet. (2022) 13:13. doi: 10.3389/fgene.2022.929477, PMID: 36061213 PMC9428447

[12] Manenti I Viola I Ala U Cornale P Macchi E Toschi P. Adaptation response in sheep: ewes in different cortisol clusters reveal changes in the expression of salivary miRNAs. Animals. (2023) 13:3273–3. doi: 10.3390/ani13203273, PMID: 37893997 PMC10603754

[13] Miretti S Lecchi C Ceciliani F Baratta M. MicroRNAs as biomarkers for animal health and welfare in livestock. Front. Vet. Sci. (2020) 7:7. doi: 10.3389/fvets.2020.578193, PMID: 33392281 PMC7775535

[14] Taxis TM Casas E. MicroRNA expression and implications for infectious diseases in livestock, CABI reviews. CABI Int. (2017) 12:1–20. doi: 10.1079/PAVSNNR201712026

[15] Fernández-García V Calvo L. Landscape implications of contemporary abandonment of extensive sheep grazing in a globally important agricultural heritage system. Land. (2023) 12:780. doi: 10.3390/land12040780

[16] Gonzales-Barron U Popova T Bermúdez Piedra R Tolsdorf A Geß A Pires J. Fatty acid composition of lamb meat from Italian and German local breeds. Small Rumin Res. (2021) 200:106384. doi: 10.1016/j.smallrumres.2021.106384

[17] Tomažin U Batorek-Lukač N Škrlep M Prevolnik-Povše M Čandek-Potokar M. Meat and fat quality of Krškopolje pigs reared in conventional and organic production systems. Animal. (2018) 13:1103–10. doi: 10.1017/s1751731118002409, PMID: 30289382

[18] Food and Agriculture Organization, The second report on the state of the world’s FAO Commision on genetic resources for food and agriculture assessments. (2015) Available at: www.fao.org/3/a-i4787e.pdf (Accessed June 06, 2024)

[19] Battaglini L Bovolenta S Gusmeroli F Salvador S Sturaro E. Environmental sustainability of alpine livestock farms. Ital J Anim Sci. (2014) 13:3155. doi: 10.4081/ijas.2014.3155

[20] Marsoner T Vigl LE Manck F Jaritz G Tappeiner U Tasser E. Indigenous livestock breeds as indicators for cultural ecosystem services: a spatial analysis within the alpine space. Ecol Indic. (2018) 94:55–63. doi: 10.1016/j.ecolind.2017.06.046

[21] Hoffmann I. Adaptation to climate change – exploring the potential of locally adapted breeds. Animal. (2013) 7:346–62. doi: 10.1017/s1751731113000815, PMID: 23739476

[22] Wildeus S. Current concepts in synchronization of estrus: sheep and goats. J Anim Sci. (2000) 77:1–14. doi: 10.2527/jas2000.00218812007700ES0040x

[23] Lynch JJ Hinch GN Adams DB. The behaviour of sheep-biological principles and implications for production. Wallingford: CAB International & CSIRO Australia (1992).

[24] Gröschl M Rauh M. Influence of commercial collection devices for saliva on the reliability of salivary steroids analysis. Steroids. (2006) 71:1097–100. doi: 10.1016/j.steroids.2006.09.007 17070563

[25] Nater UM La Marca R Florin L Moses A Langhans W Koller MM. Stress-induced changes in human salivary alpha-amylase activity—associations with adrenergic activity. Psychoneuroendocrinology. (2006) 31:49–58. doi: 10.1016/j.psyneuen.2005.05.010 16002223

[26] Topkas E Keith P Dimeski G Cooper-White J Punyadeera C. Evaluation of saliva collection devices for the analysis of proteins. Clin Chim Acta. (2012) 413:1066–70. doi: 10.1016/j.cca.2012.02.020 22405932

[27] Hellemans J Mortier G De Paepe A Speleman F Vandesompele J. qBase relative quantification framework and software for management and automated analysis of real-time quantitative PCR data. Genome Biol. (2007) 8:R19. doi: 10.1186/gb-2007-8-2-r19, PMID: 17291332 PMC1852402

[28] Bigi D Zanon A. Atlante delle razze autoctone – Bovini, equini, ovicaprini, suini allevati in Italia. 2nd ed. Italia: Edagricole (2020).

[29] Bartlewski PM Baby TE Giffin JL. Reproductive cycles in sheep. Anim Reprod Sci. (2011) 124:259–68. doi: 10.1016/j.anireprosci.2011.02.024 21411253

[30] Abecia JA Forcada F González-Bulnes A. Hormonal control of reproduction in small ruminants. Anim Reprod Sci. (2012) 130:173–9. doi: 10.1016/j.anireprosci.2012.01.011 22325928

[31] Greyling JPC Brink WCJ. Synchronization of oestrus in sheep: the use of controlled internal drug release (CIDR) dispensers. S Afr J Anim Sci. (1987) 17:128–32. doi: 10.4314/sajas.v17i3

[32] Quirke JF. Regulation of puberty and reproduction in female lambs: a review. Livest Prod Sci. (1981) 8:37–53. doi: 10.1016/0301-6226(81)90029-4

[33] Corner RA Mulvaney FJ Morris ST West DM Morel PCH Kenyon PR. A comparison of the reproductive performance of ewe lambs and mature ewes. Small Rumin Res. (2013) 114:126–33. doi: 10.1016/j.smallrumres.2013.05.018

[34] Santos-Jimenez Z Martínez-Ros P Encinas T Morales-Cruz JL Guerrero-Gallegos HZ Gonzalez-Avalos R. Ovarian response and fertility after short-term Progestagen/eCG treatments are compromised in nulliparous sheep during non-breeding season. Vet Sci. (2022) 9:663–3. doi: 10.3390/vetsci9120663, PMID: 36548824 PMC9781245

[35] Ungerfeld R. Reproductive response of mature and nulliparous yearling ewes to the ram effect during the non-breeding season. Small Rumin Res. (2016) 140:37–9. doi: 10.1016/j.smallrumres.2016.05.017

[36] Contreras-Aguilar MD Escribano D Quiles A López-Arjona M Cerón JJ Martínez-Subiela S. Evaluation of new biomarkers of stress in saliva of sheep. Animal. (2019) 13:1278–86. doi: 10.1017/S1751731118002707, PMID: 30362447

[37] Yates DT Ross TT Hallford DM Yates LJ Wesley RL. Technical note: comparison of salivary and serum cortisol concentrations after adrenocorticotropic hormone challenge in ewes. J Anim Sci. (2013) 88:599–603. doi: 10.2527/jas.2009-2204, PMID: 19854993

[38] Weaver SJ Hynd PI Ralph CR Hocking Edwards JE Burnard CL Narayan E. Chronic elevation of plasma cortisol causes differential expression of predominating glucocorticoid in plasma, saliva, fecal, and wool matrices in sheep. Domest Anim Endocrinol. (2021) 74:106503. doi: 10.1016/j.domaniend.2020.106503, PMID: 32846373

[39] Farahmandian F Chalmeh A Pourjafar M Amirian A. Linear regression between fecal cortisol metabolites and circulating cortisol levels of peri-partum ewes: suggesting a non-invasive alternative sampling method. Small Rumin Res. (2023) 227:107071. doi: 10.1016/j.smallrumres.2023.107071

[40] Jansen HT West CR Lehman MN Padmanabhan V. Ovarian estrogen receptor-β (ERβ) regulation: I. Changes in ERβ messenger RNA expression prior to ovulation in the ewe 1. Biol Reprod. (2001) 65:866–72. doi: 10.1095/biolreprod65.3.866, PMID: 11514352

[41] De Paoli M Zakharia A Werstuck GH. The role of estrogen in insulin resistance: a review of clinical and preclinical data. Am J Pathol. (2021) 191:1490–8. doi: 10.1016/j.ajpath.2021.05.011 34102108

[42] Schernthaner-Reiter MH Wolf P Vila G Luger A. The interaction of insulin and pituitary hormone syndromes. Front Endocrinol. (2021) 12:626427. doi: 10.3389/fendo.2021.626427, PMID: 33995272 PMC8113952

[43] Freitas-de-Melo A Garcia Kako Rodriguez M Crosa C Ungerfeld R. Social stress during the estrus or luteal phase in sheep. J Appl Anim Wel Sci. (2022) 27:1–9. doi: 10.1080/10888705.2021.2021408, PMID: 38314791

[44] Mayer JP Zhang F DiMarchi RD. Insulin structure and function. Biopolymers. (2007) 88:687–713. doi: 10.1002/bip.20734 17410596

[45] Wan Z Yang H Chen P Wang Z Cai Y Yao X. The novel competing endogenous long noncoding RNA SM2 regulates gonadotropin secretion in the Hu sheep anterior pituitary by targeting the oar-miR-16b/TGF-β/SMAD2 signaling pathway. Cells. (2022) 11:985. doi: 10.3390/cells11060985, PMID: 35326436 PMC8947352

[46] Pasquinelli AE Reinhart BJ Slack F Martindale MQ Kuroda MI Maller B. Conservation of the sequence and temporal expression of let-7 heterochronic regulatory RNA. Nature. (2000) 408:86–9. doi: 10.1038/35040556, PMID: 11081512

[47] Roush S Slack FJ. The let-7 family of micro RNAs. Trends Cell Biol. (2008) 18:505–16. doi: 10.1016/j.tcb.2008.07.007 18774294

[48] Hossain MM Sohel MMH Schellander K Tesfaye D. Characterization and importance of micro RNAs in mammalian gonadal functions. Cell Tissue Res. (2012) 349:679–90. doi: 10.1007/s00441-012-1469-6 22842772

[49] De Los RM Dettleff P Palomino J Peralta OA Vergara A. Dynamic expression of follicle-stimulating hormone and estrogen mRNA receptors associated with micro RNAs 34a and-let-7c in canine follicles during the estrous cycle. Animals. (2024) 14:214–4. doi: 10.3390/ani14020214, PMID: 38254383 PMC10812696

[50] Dai T Kang X Yang C Mei S Wei S Guo X. Integrative analysis of miRNA-mRNA in ovarian granulosa cells treated with Kisspeptin in Tan sheep. Animals. (2022) 12:2989–9. doi: 10.3390/ani12212989, PMID: 36359113 PMC9656243

[51] Zhang XD Zhang YH Ling YH Liu Y Cao HG Yin ZJ. Characterization and differential expression of micro RNAs in the ovaries of pregnant and non-pregnant goats (*Capra hircus*). BMC Genomics. (2013) 14:157. doi: 10.1186/1471-2164-14-157, PMID: 23497306 PMC3599660

[52] Jiajie T Yanzhou Y Hoi-Hung AC Zi-Jiang C Wai-Yee C. Conserved miR-10 family represses proliferation and induces apoptosis in ovarian granulosa cells. Sci Rep. (2017) 7:41304. doi: 10.1038/srep41304, PMID: 28112253 PMC5256277

[53] Sirotkin AV Ovcharenko D Grossmann R Lauková M Mlynček M. Identification of MicroRNAs controlling human ovarian cell steroidogenesis via a genome-scale screen. J Cell Physiol. (2009) 219:415–20. doi: 10.1002/jcp.21689, PMID: 19194990

[54] Peng JY An XP Fang F Gao KX Xin HY Han P. MicroRNA-10b suppresses goat granulosa cell proliferation by targeting brain-derived neurotropic factor. Domest Anim Endocrinol. (2016) 54:60–7. doi: 10.1016/j.domaniend.2015.09.005, PMID: 26513157

[55] Li Q Du X Pan Z Zhang L Li Q. The transcription factor SMAD4 and miR-10b contribute to E2 release and cell apoptosis in ovarian granulosa cells by targeting CYP19A1. Mol Cell Endocrinol. (2018) 476:84–95. doi: 10.1016/j.mce.2018.04.012, PMID: 29723543

[56] Wang W Ge L Zhang L Liu L Zhang X Ma X. MicroRNA-16 represses granulosa cell proliferation in polycystic ovarian syndrome through inhibition of the PI3K/Akt pathway by downregulation of Apelin 13. Hum Fertil. (2021) 26:611–21. doi: 10.1080/14647273.2021.1998661, PMID: 34854361

[57] Gad A Nemcova L Murin M Kanka J Laurincik J Benc M. micro RNA expression profile in porcine oocytes with different developmental competence derived from large or small follicles. Mol Reprod Dev. (2019) 86:426–39. doi: 10.1002/mrd.23121 30756429

[58] Li Z He X Zhang X Zhang J Guo X Sun W. Analysis of expression profiles of Circ RNA and MiRNA in oviduct during the follicular and luteal phases of sheep with two fecundity (Fec B gene) genotypes. Animals. (2021) 11:2826–6. doi: 10.3390/ani11102826, PMID: 34679847 PMC8532869

[59] Song P Yue Q Fu Q Li X Li X Zhou R. Integrated analysis of miRNA–mRNA interaction in ovaries of Turpan black sheep during follicular and luteal phases. Reprod Domest Anim. (2020) 56:46–57. doi: 10.1111/rda.13848, PMID: 33098173

[60] Pandey DP Picard D. miR-22 inhibits estrogen signaling by directly targeting the estrogen receptor α mRNA. Mol Cell Biol. (2009) 29:3783–90. doi: 10.1128/mcb.01875-08 19414598 PMC2698751

[61] Yang X Zhou Y Peng S Wu L Lin H Wang S. Differentially expressed plasma micro RNAs in premature ovarian failure patients and the potential regulatory function of mir-23a in granulosa cell apoptosis. Reproduction. (2012) 144:235–44. doi: 10.1530/rep-11-0371, PMID: 22653319

[62] Nie M Yu S Peng S Fang Y Wang H Yang X. miR-23a and miR-27a promote human granulosa cell apoptosis by targeting SMAD51. Biol Reprod. (2015) 93:98–10. doi: 10.1095/biolreprod.115.130690, PMID: 26400397

[63] Luo H Han Y Liu J Zhang Y. Identification of micro RNAs in granulosa cells from patients with different levels of ovarian reserve function and the potential regulatory function of miR-23a in granulosa cell apoptosis. Gene. (2019) 686:250–60. doi: 10.1016/j.gene.2018.11.025, PMID: 30453069

[64] Xiao-Hong H Meng W Yang-Yang P Jiang-Feng F Jing-Lei W Ling Z. Effect of follicle-stimulating hormone and luteinizing hormone on apoptosis, autophagy, and the release and reception of some steroid hormones in yak granulosa cells through miR-23a/ASK1 axis. Cell Signal. (2024) 115:111010. doi: 10.1016/j.cellsig.2023.111010, PMID: 38128707

[65] Kim EP Kim CY Heo MY Kim SW Kim GA. MicroRNA expression variation in female dog (*Canis familiaris*) reproductive organs with age and presence of Uteropathy. Animals. (2022) 12:3352–2. doi: 10.3390/ani12233352, PMID: 36496873 PMC9740207

[66] Donadeu FX Schauer SN. Differential miRNA expression between equine ovulatory and anovulatory follicles. Domest Anim Endocrinol. (2013) 45:122–5. doi: 10.1016/j.domaniend.2013.06.006, PMID: 23932580

[67] Yuan H Lu J Xiao SY Han XY Song XT Qi MY. miRNA expression analysis of the sheep follicle during the prerecruitment, dominant, and mature stages of development under FSH stimulation. Theriogenology. (2022) 181:161–9. doi: 10.1016/j.theriogenology.2022.01.001, PMID: 35101680

[68] Guelfi G Stefanetti V De Luca S Giontella A Barile VL Barbato O. Serum micro RNAs in buffalo cows: potential biomarkers of pregnancy. Res Vet Sci. (2017) 115:294–300. doi: 10.1016/j.rvsc.2017.06.001, PMID: 28628844

[69] Zhang L Zhang X Zhang X Lu Y Li L Cui S. MiRNA-143 mediates the proliferative signaling pathway of FSH and regulates estradiol production. J Endocrinol. (2017) 234:1–14. doi: 10.1530/joe-16-0488, PMID: 28649090

[70] Zhang Z Tang J Di R Liu Q Wang X Gan S. Integrated hypothalamic transcriptome profiling reveals the reproductive roles of mRNAs and miRNAs in sheep. Front Genet. (2020) 10:1296. doi: 10.3389/fgene.2019.01296, PMID: 32010181 PMC6974689

[71] Zhong Y Li L Chen Z Diao S He Y Zhang Z. MIR143 inhibits steroidogenesis and induces apoptosis repressed by H3K27me3 in granulosa cells. Front Cell Dev Biol. (2020) 8:565261. doi: 10.3389/fcell.2020.565261, PMID: 33195195 PMC7604341

[72] Zhang Z Chen CZ Xu MQ Zhang LQ Liu JB Gao Y. MiR-31 and miR-143 affect steroid hormone synthesis and inhibit cell apoptosis in bovine granulosa cells through FSHR. Theriogenology. (2019) 123:45–53. doi: 10.1016/j.theriogenology.2018.09.020, PMID: 30278258

[73] Zhang WJ Cao WY Huang YQ Cui YH Tu BX Wang LF. The role of miR-150 in stress-induced anxiety-like behavior in mice. Neurotox Res. (2018) 35:160–72. doi: 10.1007/s12640-018-9943-x, PMID: 30120712

[74] Zhou R Miao Y Li Y Li X Xi J Zhang Z. MicroRNA-150 promote apoptosis of ovine ovarian granulosa cells by targeting STAR gene. Theriogenology. (2019) 127:66–71. doi: 10.1016/j.theriogenology.2019.01.003, PMID: 30669067

[75] Geng XJ Zhao DM Mao GH Tan L. MicroRNA-150 regulates steroidogenesis of mouse testicular Leydig cells by targeting STAR. Reproduction. (2017) 154:229–36. doi: 10.1530/rep-17-0234, PMID: 28611112

[76] Du X He X Liu Q Liu Q Di R Chu M. Identification of photoperiod-induced specific miRNAs in the adrenal glands of Sunite sheep (*Ovis aries*). Front Vet Sci. (2022) 9:888207. doi: 10.3389/fvets.2022.888207, PMID: 35937294 PMC9354845

[77] Liu A Chen X Liu M Zhang L Ma X Tian S. Differential expression and functional analysis of Circ RNA in the ovaries of low and high fecundity Hanper sheep. Animals. (2021) 11:1863–3. doi: 10.3390/ani11071863, PMID: 34201517 PMC8300399

[78] Yang J Tang J He X Di R Zhang X Zhang J. Comparative transcriptomics identify key pituitary circular RNAs that participate in sheep (*Ovis aries*) reproduction. Animals. (2023) 13:2711–1. doi: 10.3390/ani13172711, PMID: 37684975 PMC10486758

[79] Gu B Liu H Han Y Chen Y Jiang H. Integrated analysis of miRNA and mRNA expression profiles in 2-, 6-, and 12-month-old small tail Han sheep ovaries reveals that oar-miR-432 downregulates RPS6KA1 expression. Gene. (2019) 710:76–90. doi: 10.1016/j.gene.2019.02.095, PMID: 30898702

[80] He H Li D Tian Y Wei Q Amevor FK Sun C. miRNA sequencing analysis of healthy and atretic follicles of chickens revealed that miR-30a-5p inhibits granulosa cell death via targeting Beclin 1. J Anim Sci Biotechnol. (2022) 13:55. doi: 10.1186/s40104-022-00697-0, PMID: 35410457 PMC9003977

[81] Zheng Y Chen K Zheng X Li H Wang G. Identification and bioinformatics analysis of micro RNAs associated with stress and immune response in serum of heat-stressed and normal Holstein cows. Cell Stress Chaperones. (2014) 19:973–81. doi: 10.1007/s12192-014-0521-8, PMID: 24917036 PMC4389857

[82] Lee J Lee S Son J Lim H Kim E Kim D. Analysis of circulating-micro RNA expression in lactating Holstein cows under summer heat stress. PLoS One. (2020) 15:e0231125. doi: 10.1371/journal.pone.0231125, PMID: 32866172 PMC7458322

[83] Cattaneo A Suderman M Cattane N Mazzelli M Begni V Maj C. Long-term effects of stress early in life on micro RNA-30a and its network: preventive effects of lurasidone and potential implications for depression vulnerability. Neurobiol Stress. (2020) 13:100271. doi: 10.1016/j.ynstr.2020.100271, PMID: 33344724 PMC7739180

[84] da Silva Rosa MP Bridi A de Ávila FG Prado CM Bastos NM Sangalli JR. Corpus luteum presence in the bovine ovary increase intrafollicular progesterone concentration: consequences in follicular cells gene expression and follicular fluid small extracellular vesicles miRNA contents. J Ovarian Res. (2024) 17:65. doi: 10.1186/s13048-024-01387-3, PMID: 38500173 PMC10946200

[85] Hughes CHK Rogus A Inskeep EK Pate JL. NR5A2 and potential regulatory miRNAs in the bovine CL during early pregnancy. Reproduction. (2021) 161:173–82. doi: 10.1530/rep-20-0009 33320828

[86] Baddela VS Onteru SK Singh D. A syntenic locus on buffalo chromosome 20: novel genomic hotspot for miRNAs involved in follicular-luteal transition. Funct Integr Genomics. (2016) 17:321–34. doi: 10.1007/s10142-016-0535-7, PMID: 27866284

[87] Yang H Fu L Li L Zhang D Li Q Zhou P. miR-665 overexpression inhibits the apoptosis of luteal cells in small ruminants suppressing HPGDS. Theriogenology. (2023) 206:40–8. doi: 10.1016/j.theriogenology.2023.04.027, PMID: 37178673

[88] Wang J Chen H Zhang Y Jiang S Zeng X Shen H. Comprehensive analysis of differentially expressed circ RNAs in the ovaries of low-and high-fertility sheep. Animals. (2023) 13:236. doi: 10.3390/ani13020236, PMID: 36670776 PMC9854751

[89] Yang H Liu X Hu G Xie Y Lin S Zhao Z. Identification and analysis of micro RNAs-mRNAs pairs associated with nutritional status in seasonal sheep. Biochem Biophys Res Commun. (2018) 499:321–7. doi: 10.1016/j.bbrc.2018.03.155, PMID: 29588175

[90] Pokharel K Peippo J Honkatukia M Seppälä A Rautiainen J Ghanem N. Integrated ovarian mRNA and miRNA transcriptome profiling characterizes the genetic basis of prolificacy traits in sheep (*Ovis aries*). BMC Genomics. (2018) 19:104–17. doi: 10.1186/s12864-017-4400-4, PMID: 29378514 PMC5789708

[91] Zhai M Xie Y Liang H Lei X Zhao Z. Comparative profiling of differentially expressed micro RNAs in estrous ovaries of Kazakh sheep in different seasons. Gene. (2018) 664:181–91. doi: 10.1016/j.gene.2018.04.025, PMID: 29704632

[92] Ullah Y Li C Li X Ni W Yao R Xu Y. Identification and profiling of pituitary microRNAs of sheep during anestrus and estrus stages. Animals. (2020) 10:402. doi: 10.3390/ani10030402, PMID: 32121341 PMC7142988

