# Supplementary material for: Expression profiles of circulating miRNAs in an endangered Piedmontese sheep breed during the estrus cycle
Source: Front Vet Sci. 2024 Nov 5;11:1458463. doi: 10.3389/fvets.2024.1458463 (PMC11575464; doi:10.3389/fvets.2024.1458463)
Supplement: Supplementary file 1 [file Data_Sheet_1.docx]

| *Behavior* | *Description* |
| --- | --- |
| **Aggressive and threatening behaviors** | |
| Butt | Sudden, strong head contact with another ewe |
| Head clash | A sheep separates before loading another specimen, then colliding with the head or horns. |
| Blocking | Two animals’ head-to-head opposites begin a series of blows and headboards |
| Threaten | Directing the forehead towards another ewe with no physical contact |
| Horn threat (jerk) | Sudden downward movement of the head towards the opponent |
| Threat kick | Upward movement of the stiffened front limb that can be repeated several times with possible contact with the opponent (between males) |
| Horn and shoulder pushing | Pushed with horns and shoulders with no apparent social purpose |
| **Submission behaviors** | |
| Low neck | Lowering of the head and neck |
| **Individual behaviors** | |
| Resting | Lying on floor, either ruminating or not |
| Exploring | Nose interaction with the enclosure wall of other physical issue |
| Feeding | Standing by the feeder, with the head completely inside one of the feeder holes |
| Foraging | Standing, with the head down, interacting with the floor bedding with the mouth |
| Running | Fear reaction |
| Moving | Change position within the enclosure, either walking or running |
| Standing | Stand with the four feet on the floor, either ruminating or not |
| Self-grooming | Groom, either by self-licking or by rubbing against a physical enclosure object |
| **Social and affiliative behaviors** | |
| Pushing | Press the head against another ewe to force the pass |
| Displacing from resources | Force another ewe to leave the feeder, drinker or the mineral resource |
| Nudging | Slightly, gently push another ewe |
| Sniffing | Smell another ewe without physical contact |
| Nose | Slightly contact another ewe with the nose |
| Grooming | Clean the wool of another ewe with the mouth |
| Licking | Lick any part of another ewe’s body |
| Mount | Between males it means dominance; between females and castrated males it means game |
| Kick | Upward movement of the stiffened front limb that can be repeated several times with possible contact with another animal |
| **Sexual female behaviors** | |
| Squat/crouch | Squatting posture, usually includes urinating and it happens after a weak blow from the ram |
| Circling | The sheep turns towards the ram, often rubbing its nose on his flank; the ram usually goes behind the sheep |
| Tail fanning | The sheep's tail is raised and waved in the presence of the ram |
| Head turning | The sheep turns its head towards the male while he does approach the mount |
| Standing mount | The sheep stands firmly while the male mounts her |
| Following/migration | The sheep follows a ram after initial contact with him, often together with another sheep |
| **Sexual male behaviors** | |
| Sexual sniffing/nose | The ram smells the urine and the perianal region of the sheep |
| Flehemen reflex | After sniffing, he arches his head upwards and curls the upper lip showing teeth |
| Low stretching | Neck stretch that is held parallel to the ground with mouth forward and up. The head often rotates 90° |
| Sexual nudging | It consists of one or a combination of strokes, rubs and elongations |
| Sexual licking | the ram licks the side of the sheep |
| Pre mount | The ram is positioned behind the sheep and moves the pelvis giving small strokes; generally, it can precede the mount |
| Sexual mount | The ram's breast in close contact with the sheep's rump |
| Ejaculation | Pelvic thrust during the mount accompanied by a rapid backward movement of the head |
| **Play behaviors** | |
| Gambole | The animal jumps, commonly with speed, and twists its body and head at the same time |
| Chasing | One animal is pursued by another in an effort to reach or pass it, usually by running at medium speed |
| Reciprocal butt | Lunge with the forehead against the forehead of another animal. It is a mutual action, where both lambs participate. The behavior was often first preceded by head lowering and pawing the ground with forelegs a couple of times. |
| Jumping | The animal elevates the body from the ground several times in a row, through a springing motion by the legs. The body is rather stiff, and no twists are included. The behavior is commonly preceded by a some running. |
| Frontal butt (one-way butt) | The animal lunges with the forehead against another animal, but here it is not mutual; the other animal does not actively participate. The animal lunges against the front of the other animal. Here as well the behaviour was often preceded by head lowering and pawing. |
| Side/rear butt (one-way butt) | Same as above, except from that lunges are instead directed to the side or rear of the other animal. |
| Pivot | A quick, stiff, rotation horizontally through a little jump on the same spot. This makes the animal face another direction. |
| Racing | Running in a group, commonly back and forth in the same area repeatedly. It involves at least 3 participants and occasionally ewes join in. |

**Supplementary table 1**. Ethogram of *Ovis aries* adapted from Lynch et al., 1992


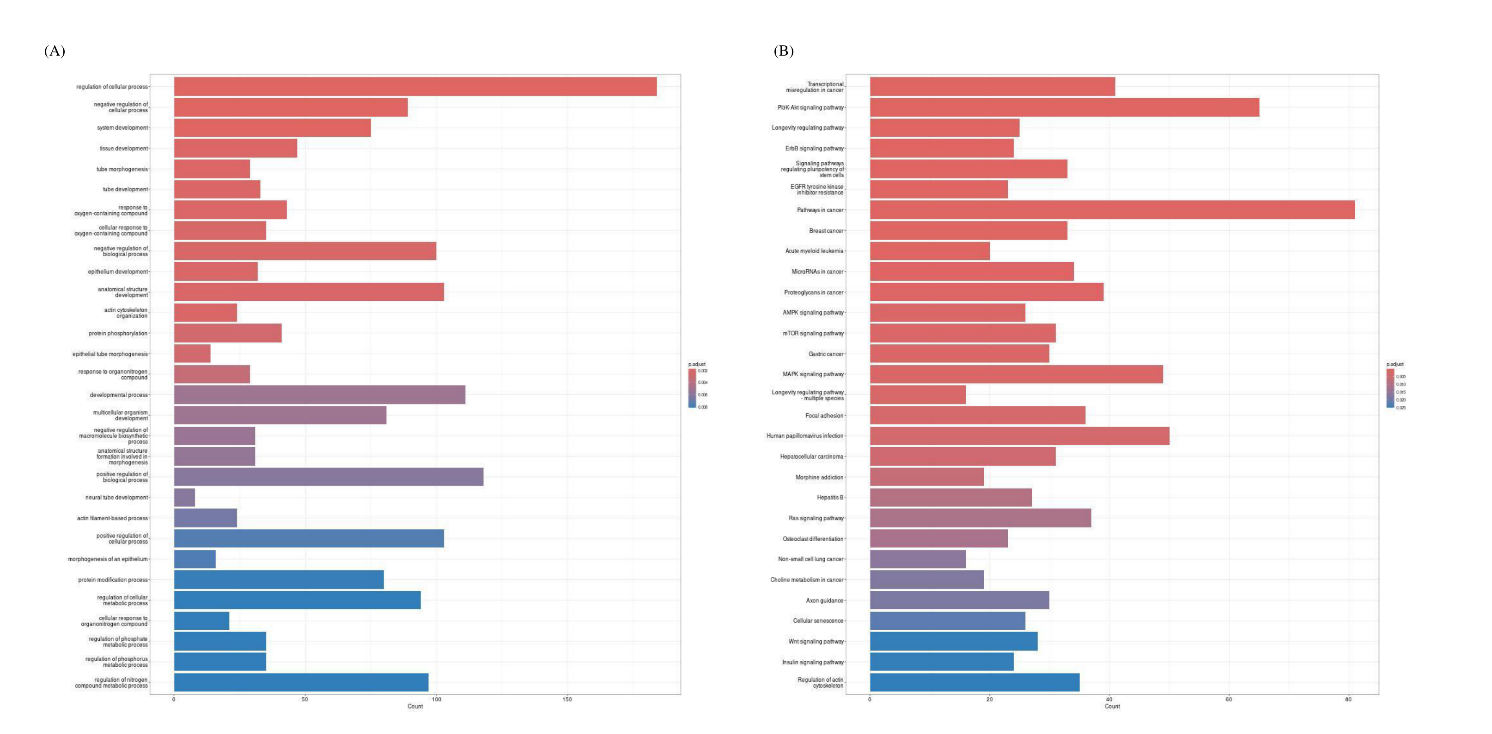


**Supplementary Figure 1.** Bar plots of pathways resulted from enrichment analyses of *Bos taurus* orthologues miRNAs downregulated: **(A)** Biological Processes in GO database, **(B)** KEGG database.


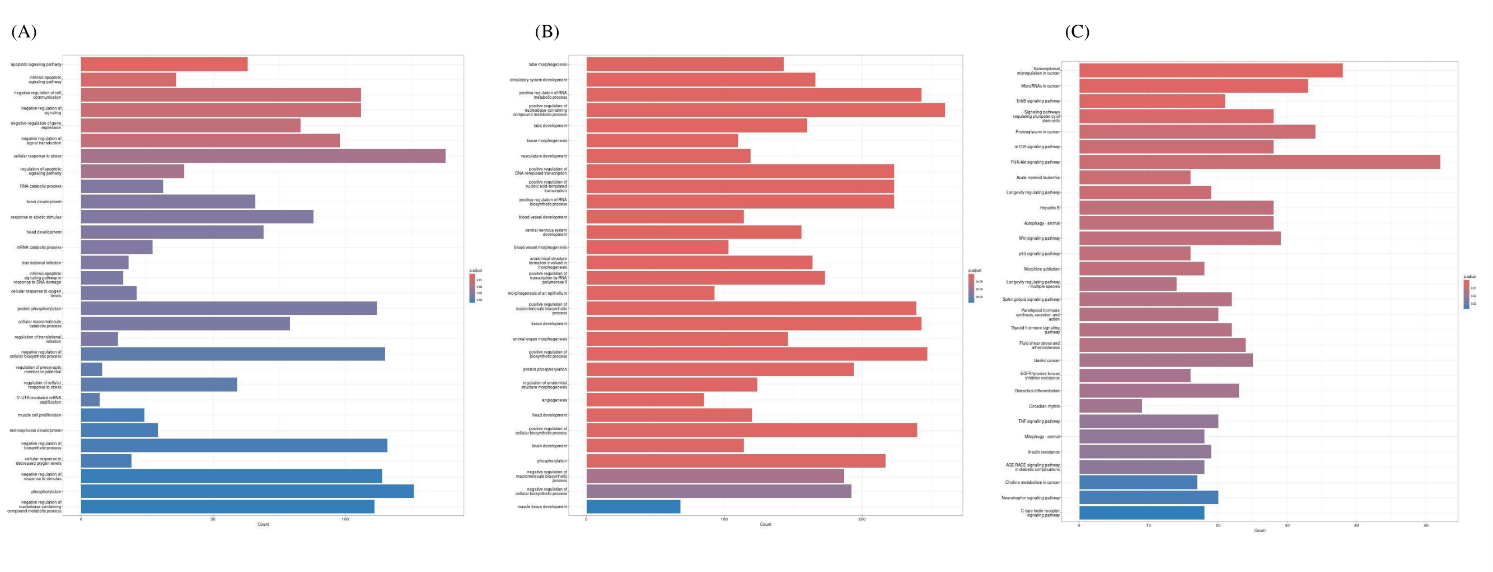


**Supplementary Figure 2.** Bar plot of pathways resulted from enrichment analyses of *Homo sapiens* orthologues miRNAs upregulated and downregulated: **(A)** upregulated, Biological Processes in GO database, **(B)** downregulated, Biological Processes in GO database, **(C)** downregulated, KEGG database.
